# Clinical and molecular spectrum of inherited epidermolysis bullosa in a Thai cohort: A 12-year retrospective study

**DOI:** 10.1016/j.jdin.2026.02.010

**Published:** 2026-03-07

**Authors:** Chavalit Supsrisunjai, Nuanjutha Tirachaimongkol, Thareena Bunnag, Nuttaporn Sengtae, Sissades Tongsima, Chumpol Ngamphiw, Wanchanida Komkhong, Tanawatt Kootiratrakarn, Vesarat Wessagowit, Piranit Kantaputra

**Affiliations:** a Department of Medical Services, Institute of Dermatology, Ministry of Public Health, Bangkok, Thailand; b Division of Pediatric Dentistry, Department of Orthodontics and Pediatric Dentistry, Faculty of Dentistry, Chiang Mai University, Chiang Mai, Thailand; c Center of Excellence in Medical Genetics Research, Faculty of Dentistry, Chiang Mai University, Chiang Mai, Thailand; d National Center for Genetic Engineering and Biotechnology, National Science and Technology Development Agency (NSTDA), Thailand Science Park, Pathum Thani, Thailand

**Keywords:** COL7A1, dermatogenetics, epidemiology, epidermolysis bullosa, genodermatoses, genotype-phenotype correlation, Thailand, whole-exome sequencing

*To the Editor:* Inherited epidermolysis bullosa (EB) comprises a group of rare genetic disorders characterized by mucocutaneous fragility and trauma-induced blistering.^1^ While the clinical and molecular spectrum of EB has been extensively described in North America and Europe, molecular data from Thailand remain limited,^2^ restricting region-specific diagnostic precision and genetic counseling. We describe the clinical features and molecular findings of a Thai EB cohort and evaluate genotype-phenotype correlations.

A retrospective study was conducted at the Institute of Dermatology, Bangkok, between January 2012 and December 2023. Patients with a clinical diagnosis of EB were included. Demographic data and clinical manifestations were extracted from medical records. Whole-exome sequencing (WES) was performed for all participants, and variants were classified according to the American College of Medical Genetics and Genomics criteria. Associations between genotype and clinical features were assessed using Fisher’s exact test, with *P* < .05 considered statistically significant. Institutional approval and informed consent were obtained.

Thirty-five patients met diagnostic criteria (mean age 22.5 years; 62.9% female). Dystrophic epidermolysis bullosa (DEB) was the most frequent subtype (65.7%), including dominant DEB (*n* = 11), recessive DEB (*n* = 8), and EB pruriginosa (*n* = 4). The distribution of causative genes, EB subtypes, and clinical features is summarized in Table I. Pathogenic or likely pathogenic variants were identified in *COL7A1*, *KRT14*, *KRT5*, *FERMT1*, *ITGB4*, and *LAMB3*, with *COL7A1* variants detected in 22 patients (Supplematary Table I, available via Mendeley at https://data.mendeley.com/datasets/dcn48vy233/1). Sixteen variants were novel, including *FERMT1* c.1139+5G>A in patients with severe Kindler syndrome, expanding the mutational spectrum in Asian populations.

**Table I:** Descriptive distribution of causative genes, epidermolysis bullosa subtypes, and clinical features in the Thai cohort

Gene	Patients, *n* (%)	EB subtype(s)	Inheritance	Key cutaneous features	Key extracutaneous features
*COL7A1*	22 (62.9)	DDEB, RDEB, EB pruriginosa	AD/AR	Blistering, scarring, milia, nail dystrophy, pruritus	Dental caries, mucosal erosions
*KRT14*	4 (11.4)	EBS	AD	Localized blistering, minimal scarring	None prominent
*KRT5*	3 (8.6)	EBS	AD	Localized trauma-induced blistering	None
*FERMT1*	2 (5.7)	Kindler syndrome	AR	Poikiloderma, photosensitivity, skin atrophy	Mucosal fragility
*ITGB4*	2 (5.7)	JEB	AR	Blistering, nonscarring alopecia, subungual hyperkeratosis	Enamel hypoplasia
*LAMB3*	1 (2.9)	JEB	AR	Severe erosions	Enamel hypoplasia, mucosal erosions

Values are shown as number (percentage of total cohort, *n* = 35).

*AD*, Autosomal dominant; *AR*, autosomal recessive; *DDEB*, dominant dystrophic epidermolysis bullosa; *DEB*, dystrophic epidermolysis bullosa; *EB*, epidermolysis bullosa; *EBS*, epidermolysis bullosa simplex; *JEB*, junctional epidermolysis bullosa; *RDEB*, recessive dystrophic epidermolysis bullosa.

Distinct genotype-phenotype correlations were observed. Gene-specific genotype-phenotype associations are presented in Fig 1. *COL7A1* variants were significantly associated with severe pruritus, scarring, milia, and nail dystrophy, consistent with classic DEB and EB pruriginosa.^3^ *KRT14* variants were associated with a lower frequency of pruritus, suggesting a comparatively attenuated inflammatory profile in epidermolysis bullosa simplex. The association of *ITGB4* variants with nonscarring alopecia and subungual hyperkeratosis broadens the phenotypic spectrum of junctional EB and highlights β4-integrin’s developmental role in hair and nail morphogenesis.^4^ Residual integrin function may permit normal appendage formation with postnatal fragility, enabling improved diagnosis, prognostication, and targeted genetic testing beyond traditionally lethal models.^4^ The single *LAMB3* case demonstrated enamel hypoplasia and mucosal erosions (*P* < .05), consistent with junctional EB and highlighting LAMB3’s role in amelogenesis, the multisystem burden of laminin-332 deficiency. Collectively, these findings indicate that characteristic clinical constellations may guide targeted genetic testing where WES access is limited.

**Fig 1:**
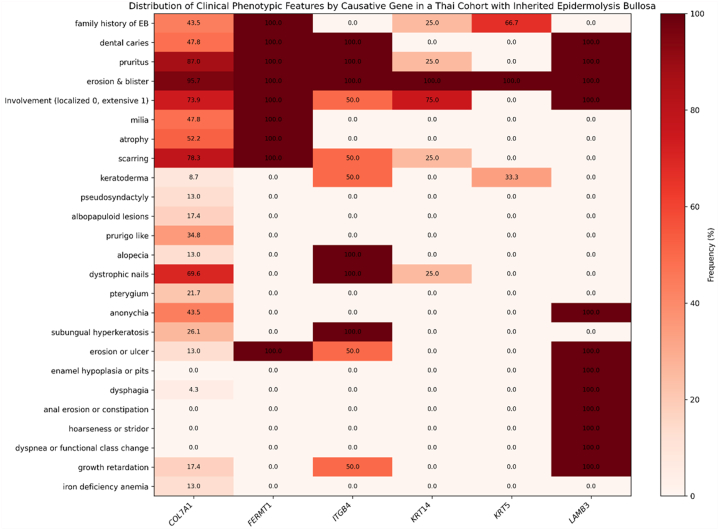
Heatmap showing the frequency (%) of clinical phenotypic features according to causative gene in Thai patients with inherited epidermolysis bullosa. Percentages are displayed within cells (0-100 scale), with darker shading indicating higher frequency.

The predominance of DEB and *COL7A1* variants parallels reports from Japan and Korea but differs from Western cohorts with higher proportions of epidermolysis bullosa simplex, underscoring the value of region-specific variant databases.^2^^,^^5^ The identification of previously unreported variants in *COL7A1*, *ITGB4*, *LAMB3*, and *FERMT1* suggests possible founder effects or under-recognized population-specific alleles in Thailand. Although functional validation was beyond the study scope, segregation data and *in silico* analyses support pathogenicity. Study limitations include the single-center, retrospective design and potential under-reporting of extracutaneous features. Nonetheless, systematic phenotyping combined with comprehensive WES provides clinical and molecular characterization of EB in Thailand and supports the integration of phenotype-driven assessment with molecular testing to improve diagnostic equity and enable future precision-therapy strategies.

### Declaration of generative AI and AI-assisted technologies in the writing process

During the preparation of this work the authors used ChatGPT-5.0 in order to check English grammar. After using this tool, the authors reviewed and edited the content as needed and take full responsibility for the content of the publication.

## Conflicts of interest

None disclosed.
